# Metastatic Testicular Choriocarcinoma: An Unusual Cause of Upper Gastrointestinal Bleed

**DOI:** 10.7759/cureus.5243

**Published:** 2019-07-26

**Authors:** Abdelkader Chaar, Jason A Mouabbi, Ahmed Alrajjal, Mohammed Barawi

**Affiliations:** 1 Internal Medicine, Ascension Saint John Hospital, Detroit, USA; 2 Hematology/oncology, Ascension Saint John Hospital, Detroit, USA; 3 Pathology, Ascension Saint John Hospital, Detroit, USA; 4 Gastroenterology, Ascension Saint John Hospital, Detroit, USA

**Keywords:** testicular choriocarcinoma, upper gi bleed, gastric metastases

## Abstract

Testicular cancer is the most common neoplasia in men between the ages of 15 to 44 years. Choriocarcinoma represents less than 2% of testicular tumors. It is usually characterized by an early hematogenous spread to the lungs and brain. Metastases to the gastrointestinal (GI) tract are extremely rare. Most metastatic lesions in the GI tract are seen in the small bowel.

We present a 30-year-old African American male with a past medical history significant for stage III non-seminomatous germ cell testicular cancer. The patient was initially started on chemotherapy; however, he was not compliant with his treatment. One year following his diagnosis, he presented to the hospital with shortness of breath and chest pain. CT angiography of the chest was done and showed multiple masses scattered in all lung fields. The lesions were believed to be metastatic in nature. Laboratory testing showed a human chorionic gonadotropin beta level of 40,453 IU/L, LDH 258 IUnits/L, and alfa-fetoprotein 8.9 ng/mL. His hospital stay was complicated with melena and a drop in his hemoglobin from a baseline of 12 to 7 gm/dL. An esophagogastroduodenoscopy (EGD) showed three erythematous friable nodules in the gastric body. Biopsy results came back consistent with metastatic choriocarcinoma. The patient was offered salvage chemotherapy; however, he refused treatment and elected to proceed with suppurative measures.

Testicular choriocarcinomas are the most aggressive and rapidly arising germ cell tumors. By the time they are diagnosed, large subsets of cases have already metastasized. Patients usually present with symptoms of hemorrhage in metastatic sites due to the high level of vascularization of those lesions. Gastrointestinal metastases from choriocarcinomas are very rare which account for 5% of all metastatic lesions with around 1% affecting the stomach. The presenting symptoms of stomach metastases are melena and/or hematemesis along with anemia.

Although extremely rare, gastric metastases of choriocarcinoma should be kept in mind as part of the differential diagnosis for young patients with upper GI bleeding.

## Introduction

Testicular cancer is the most common neoplasia in men between the ages of 15 to 44 years [1, 2]. It is estimated that 9560 new cases of testicular cancer will be diagnosed in 2019 in the United States [3]. Choriocarcinoma represents less than 2% of testicular tumors [4]. It is usually characterized by an early hematogenous spread to the lungs and brain [5]. Metastases to the gastrointestinal (GI) tract are extremely rare with most lesions involving the small bowel. Metastases to the stomach are rarely seen and only reported in a few case reports [6].

## Case presentation

We present a 30-year-old African American male with a past medical history significant for stage III non-seminomatous germ cell testicular cancer. The diagnosis was made via inguinal lymph node biopsy followed by right orchiectomy. The patient was initially started on chemotherapy with etoposide, cisplatin, and bleomycin; however, he was not compliant with his treatment. One year following his diagnosis, he presented to the hospital with dyspnea and right-sided chest pain. CT angiography of the chest was done and showed multiple masses scattered in all lung fields with the largest being in the midlung measuring 2.6 x 2.5 cm. Those lesions were believed to be metastatic in nature. At that time his human chorionic gonadotropin (hCG) beta was 40,453 IU/L, lactate dehydrogenase (LDH) 258 IUnits/L and alfa-fetoprotein 8.9 ng/mL.

During his hospital stay, he experienced melena and an abrupt fall of his hemoglobin from a baseline of 12 gm/dL to 7 gm/dL. He was given two units of packed red blood cells and started on pantoprazole. An esophagogastroduodenoscopy (EGD) was performed and showed an ulcer in the duodenal bulb with a visible bleeding vessel. Two endoclips were placed and 6 cubic centimeters of 1:20,000 epinephrine solution was injected to achieve hemostasis. His hemoglobin remained stable and he was subsequently discharged home on oral omeprazole.

**Figure 1:**
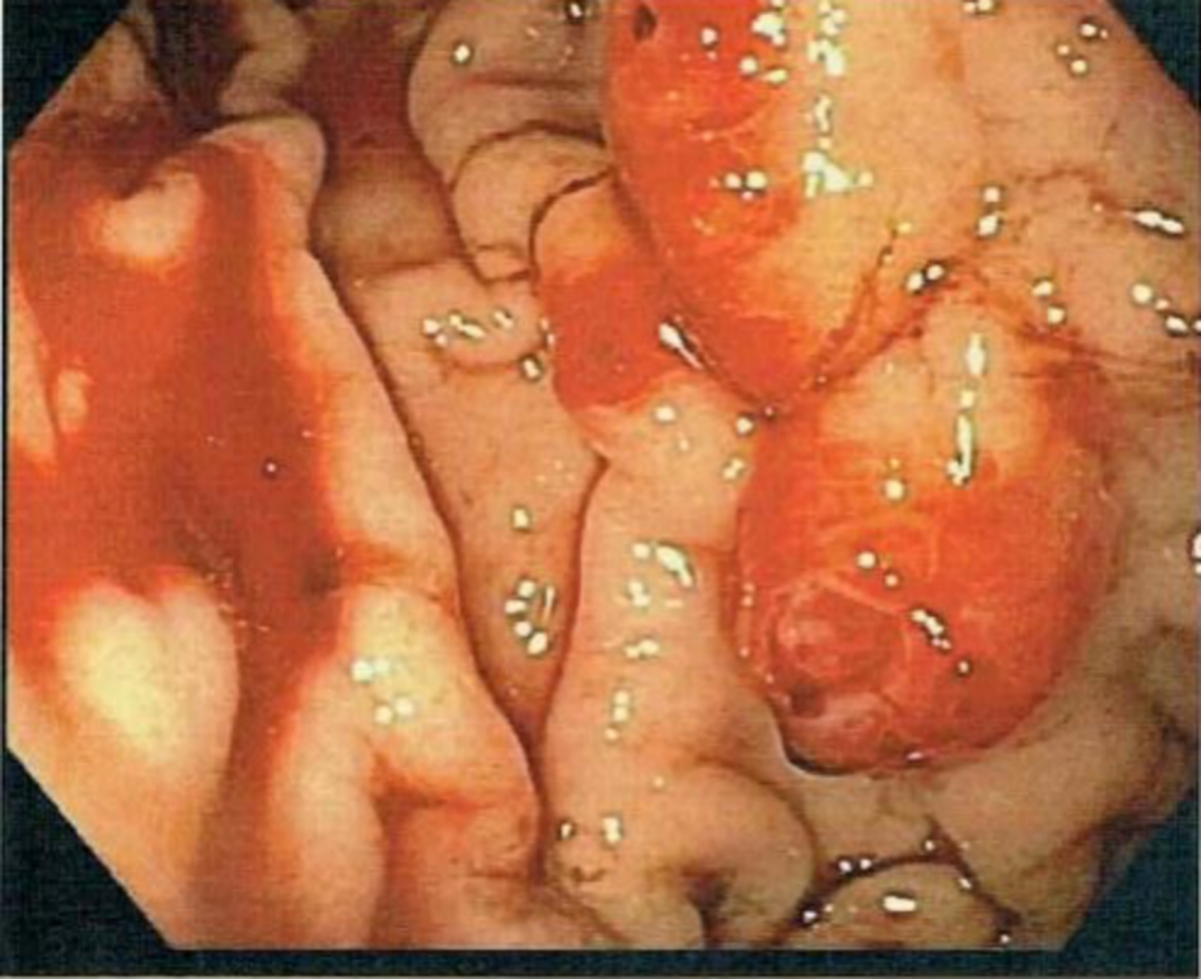
Erythematous friable nodules found in the gastric body

Two weeks later, he presented to the emergency department with generalized weakness and black tarry stool. On admission, his hemoglobin was 4.5 gm/dL. An EGD at this time showed three erythematous friable nodules in the gastric body (Figure 1). These lesions were not seen on the previous EGD.

Spontaneous oozing was noted from these lesions upon touching them. Hemostasis was eventually achieved using epinephrine injection along with the argon plasma coagulation system to ablate the oozing spots.

**Figure 2:**
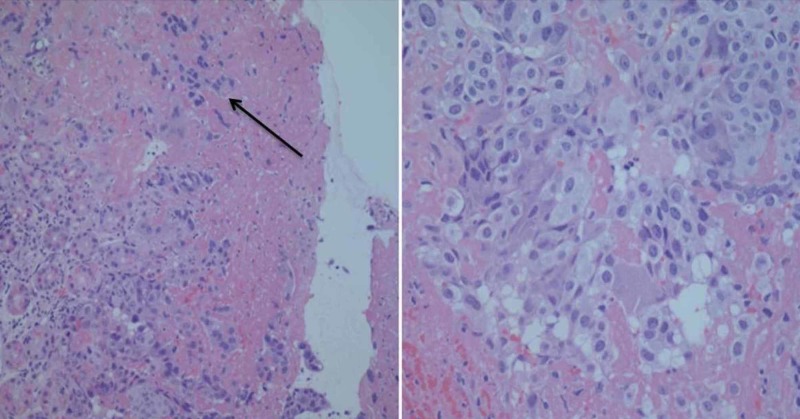
Histological examination showing gastric mucosa with surface ulceration and infiltration of cytotrophoblast rimmed with syncytiotrophoblast (arrow)

Histological examination of the lesions tissue biopsy showed both cytotrophoblast and syncytiotrophoblast and diagnosis of gastric metastases of choriocarcinoma was subsequently made (Figure 2).

The patient was offered salvage chemotherapy; however, he refused treatment and was elected to proceed with suppurative measures.

## Discussion

Testicular choriocarcinomas are the most aggressive and rapidly arising germ cell tumors. At the time they are diagnosed, a large subset of cases have already metastasized making the initial presentation mostly with metastasis-related symptoms [5]. Patients usually present with symptoms of hemorrhage in metastatic sites due to the high level of vascularization of those lesions. Since the most common metastatic sites are the lungs and the brain; patients usually present with seizures, stroke-like symptoms, confusion and/or hemoptysis.

Diagnosis of primary choriocarcinoma is done by radical inguinal orchiectomy to allow histologic evaluation. The serum concentration of human chorionic gonadotropin beta is used to monitor response to treatment. According to the International Germ Cell Cancer Collaboration group, a level higher than 50,000 IU/L puts the patient in a poor prognostic group [7].

Although testicular cancer has one of the highest cure rates of all cancers with an average five-year survival rate of 95% [8], choriocarcinoma is not as sensitive to chemotherapy. Most cases are so advanced on the presentation that patients don’t respond to standard chemotherapy which includes three or four cycles of BEP (Bleomycin, etoposide, and cisplatin) [9]. Salvage chemotherapy in relapsing cases with vinblastine and ifosfamide may help in reducing the tumor burden however this subset of patients may end up on palliative measures as their only option.

Gastrointestinal metastases from testicular tumors are very rare and account for 5% of all metastatic lesions; 1% involve the stomach [10]. The presence of gastric metastases is an important indicator of advanced disease at the time of detecting these lesions [11]. Presenting symptoms of stomach metastases may include melena and/or hematemesis along with anemia [12].

## Conclusions

Although extremely rare, gastric metastases of choriocarcinoma should be kept in mind as part of the differential diagnosis for young patients with upper gastrointestinal bleeding.
